# Housing Insecurity and the Association With Health Outcomes and Unhealthy Behaviors, Washington State, 2011

**DOI:** 10.5888/pcd12.140511

**Published:** 2015-07-09

**Authors:** Mandy Stahre, Juliet VanEenwyk, Paul Siegel, Rashid Njai

**Affiliations:** Author Affiliations: Juliet VanEenwyk (retired), Non-Infectious Conditions Epidemiology, Washington State Department of Health, Olympia, Washington; Paul Siegel, Rashid Njai, National Center for Chronic Disease Prevention and Health Promotion, Centers for Disease Control and Prevention, Atlanta, Georgia. Dr Stahre is also affiliated with the Office of Surveillance, Epidemiology, and Laboratory Services, Centers for Disease Control and Prevention, Atlanta, Georgia.

## Abstract

Few studies of associations between housing and health have focused on housing insecurity and health risk behaviors and outcomes. We measured the association between housing insecurity and selected health risk behaviors and outcomes, adjusted for socioeconomic measures, among 8,415 respondents to the 2011 Washington State Behavioral Risk Factor Surveillance System. Housing insecure respondents were about twice as likely as those who were not housing insecure to report poor or fair health status or delay doctor visits because of costs. This analysis supports a call to action among public health practitioners who address disparities to focus on social determinants of health risk behaviors and outcomes.

## Objective

In 2012, an estimated 41 million US households paid more than 30% of their pre-tax income for housing (1). High housing costs make it difficult to afford other necessities, including food, transportation, and medical care. Housing affordability is associated with housing insecurity or stress related to affording rent or mortgage (2,3). Studies have reported associations between housing insecurity and mental health problems or avoiding medical care, but questions remain about the association with health risk behaviors and outcomes (4–6). This study characterizes adults who report housing insecurity and the relationship of housing insecurity to selected unhealthy behaviors and outcomes.

## Methods

We analyzed data from the 2011 Washington State Behavioral Risk Factor Surveillance System (BRFSS). BRFSS is a random-digit–dialed telephone survey conducted annually in all 50 states, DC, and US territories. The Washington State BRFSS response rate for 2011 was about 47%. Data from 8,415 respondents responding to the state-added Social Context Module were used to assess the frequency of housing insecurity, which was defined as respondents answering “always,” “usually,” or “sometimes” to “How often in the past 12 months would you say you were worried or stressed about having enough money to pay your rent/mortgage?” (6), and the associations between housing insecurity and health risk behaviors and outcomes. We calculated unadjusted prevalence estimates with 95% confidence intervals (CIs) for housing insecurity, stratified by socioeconomic measures and demographics. Categorical variables representing socioeconomic and demographic measures were educational level, income (when available), home ownership, sex, health insurance coverage, Hispanic ethnicity, age, marital status, veteran status, presence of children in the home, and self-report of experiencing 3 or more adverse childhood experiences (ACEs) (eg, physical abuse) from 11 questions included in the state-added ACE module. Unadjusted prevalence ratios (PRs), PRs adjusted for socioeconomic measures and demographics (aPRs), and 95% CIs using predicted marginals were estimated to assess the relationship between housing insecurity and the following measures: current smoking, binge drinking during the past 30 days (defined as consuming 5 or more drinks on an occasion for men and 4 or more drinks on an occasion for women), delaying doctor visits because of costs in the last year, poor or fair self-reported health status, as well as 14 days or more in the past 30 days of poor physical health, poor mental health, or poor health limiting daily activity. These health risk behaviors and outcomes were chosen as a sample of quality of life indicators that are associated with different types of stressful events. All estimates used Washington State–specific raked and trimmed weights and were performed using SUDAAN version 11 (RTI International) to account for sampling weights and to adjust variance estimates for the complex sampling design.

## Results

Among all Washington respondents, 29.4% reported housing insecurity. Respondents with the following characteristics reported a prevalence of housing insecurity higher than the state prevalence: high school education or less, annual household income less than $50,000, women, Hispanic ethnicity, aged 25 to 44 years, unmarried, living in households with children, or 3 or more ACEs (Table 1). The groups with the highest frequency of always being worried or stressed about having enough money to pay their rent or mortgage were respondents with incomes less than $25,000, people without health insurance at the time of survey, renters, and people with a self-reported history of 3 or more adverse childhood experiences.

**Table 1:** Frequency of Housing Insecurity in the Past 12 Months by Selected Measures of Socioeconomic Status and Demographics, Washington State, 2011

Socioeconomic Status	Housing Insecure			
	Always, % (95% CI)	Usually, % (95% CI)	Sometimes, % (95% CI)	Rarely/Never, % (95% CI)
**Education**				
High school graduate or less	10.4 (8.4–12.8)	6.1 (4.6–8.1)	21.8 (19.1–24.7)	61.7 (58.4–64.9)
Some college	6.4 (5.1–8.1)	4.9 (3.9–6.2)	18.9 (16.8–21.2)	69.7 (67.1–72.2)
College graduate	3.3 (2.5–4.3)	2.9 (2.2–3.8)	12.6 (11.0–14.4)	81.2 (79.1–83.1)
**Income, $**				
<25,000	21.2 (17.7–25.2)	7.7 (6.0–9.9)	28.1 (24.3–32.2)	43.0 (38.9–47.2)
25,000 to <50,000	6.8 (5.0–9.2)	7.7 (5.9–10.0)	22.8 (20.1–25.7)	62.8 (59.4–66.0)
50,000 to <75,000	2.7 (1.7–4.1)	3.6 (2.5–5.4)	17.5 (14.3–21.2)	76.2 (72.4–79.7)
≥75,000	1.0 (0.4–1.4)	1.2 (0.7–1.9)	10.4 (8.6–12.5)	87.6 (85.4–89.5)
**Home ownership**				
Own	4.2 (3.4–5.1)	3.6 (2.9–4.4)	15.4 (14.0–16.8)	76.9 (75.3–78.5)
Rent	16.3 (13.3–19.7)	8.8 (6.9–11.3)	27.1 (23.7–30.8)	47.8 (43.8–51.7)
**Demographics**				
**Sex**				
Male	5.5 (4.4–6.9)	3.8 (2.9–4.9)	15.3 (13.4–17.3)	75.4 (73.1–77.6)
Female	7.9 (6.6–9.5)	5.5 (4.5–6.7)	20.2 (18.4–22.2)	66.4 (64.1–68.5)
**Have health insurance at time of survey (age 18–65 y)**				
Yes	6.3 (5.3–7.5)	4.3 (3.5–5.4)	17.8 (16.2–20.0)	71.5 (69.5–73.4)
No	16.7 (12.4–22.0)	11.1 (8.2–14.8)	31.1 (26.1–36.5)	41.2 (35.8–46.8)
**Hispanic ethnicity^a^**				
Yes	5.5 (2.5–11.6)	6.9 (4.2–11.2)	27.6 (22.0–34.1)	60.0 (52.8–66.7)
No	6.9 (6.0–8.0)	4.6 (3.8–5.4)	17.2 (15.9–18.6)	71.3 (69.7–72.9)
**Age group, y**				
18–24	5.0 (2.5–9.9)	4.8 (1.9–11.7)	18.3 (12.3–26.4)	71.9 (63.0–79.4)
25–34	12.9 (8.6–19.0)	7.4 (5.1–10.8)	24.4 (19.6–30.0)	55.2 (48.9–61.4)
35–44	8.1 (6.2–10.6)	6.0 (4.1–8.5)	23.8 (20.37–27.7)	62.1 (57.9–66.1)
45–54	8.3 (6.2–11.0)	5.2 (3.8–7.1)	19.1 (16.4–22.2)	67.4 (63.8–70.8)
55–64	6.0 (4.8–7.6)	4.4 (3.3–5.7)	15.6 (13.4–18.0)	74.0 (71.3–76.6)
≥65	2.4 (1.8–3.2)	2.1 (1.6–2.8)	10.4 (9.1–12.0)	85.1 (83.3–86.7)
**Married**				
Yes	4.0 (3.3–5.0)	3.7 (3.0–4.6)	16.7 (15.2–18.4)	75.5 (73.7–77.2)
No	11.3 (9.3–13.5)	6.3 (4.9–7.9)	19.9 (17.6–22.3)	62.6 (59.6–65.5)
**Veteran status**				
Yes	4.7 (3.2–6.9)	3.5 (2.4–5.2)	11.2 (8.9–14.1)	80.5 (77.1–83.5)
No	7.1 (6.1–8.3)	4.9 (4.1–5.8)	19.0 (17.5–20.5)	69.0 (67.2–70.7)
**Children present**				
Yes	7.3 (5.8–9.1)	6.8 (5.3–8.7)	22.9 (20.5–25.6)	63.0 (60.0–65.9)
No	6.6 (5.4–7.9)	3.6 (2.9–4.3)	15.2 (13.7–16.8)	74.7 (72.8–76.5)
**ACEs**				
<3	4.1 (3.3–5.1)	3.4 (2.7–4.1)	16.3 (14.9–17.9)	76.2 (74.5–77.9)
≥3	15.1 (12.6–18.0)	8.5 (6.6–11.0)	22.8 (20.0–25.9)	53.6 (50.1–57.0)
Overall	6.8 (5.9–7.9)	4.7 (4.0–5.5)	17.9 (16.6–19.3)	70.5 (68.9–72.1)

Abbreviations: ACEs, adverse childhood experiences; CI, confidence interval.

a People identified as Hispanic can be of any race.

We categorized the frequency of housing insecurity into those who were housing insecure (reported being always, usually, or sometimes worried about making housing payments) and those who were housing secure (reported never or rarely worried). Among people reporting housing insecurity, 33.3% also reported delaying doctor visits because of costs, 26.9% were current smokers, and 26.3% had poor or fair health (Table 2). People who were housing insecure were more likely to be current smokers than people who were not insecure (aPR = 1.4). Binge drinking in the past 30 days was not significantly associated with housing insecurity. Those who were housing insecure were nearly 6 times as likely as those who were not insecure to delay doctor visits because of costs (PR = 5.7). This association was attenuated but still significant after adjusting for socioeconomic measures and demographics (aPR = 2.6).

**Table 2:** Percentage and Prevalence Ratio of Being Housing Insecure Compared With Not Being Housing Insecure by Selected Adverse Health Behaviors and Outcomes, Washington State, 2011

Health Risk Behaviors	Housing Insecure^a^		Prevalence Ratio (95% CI)		
	Yes	No	Unadjusted	Adjusted for SES^b^	Adjusted for SES and Demographics^c^
Current smoker	26.9	9.8	2.8 (2.3–3.3)	1.8 (1.5–2.2)	1.4 (1.1–1.7)
Past 30-day binge drinker	16.8	15.0	1.1 (0.9–1.4)	1.1 (0.9–1.4)	0.9 (0.8–1.1)
Delayed doctor visit because of costs	33.3	5.9	5.7 (4.7–6.8)	4.0 (3.2–4.9)	2.6 (2.1–3.3)
**Health outcomes**					
**Poor/fair health status**	26.3	11.3	2.3 (2.0–2.7)	1.5 (1.3–1.8)	1.9 (1.5–2.4)
**≥14 days in the past 30 days**					
Poor health limiting daily activity	14.3	5.0	2.9 (2.3–3.6)	2.0 (1.6–2.5)	2.0 (1.5–2.6)
Poor physical health	17.4	8.4	2.1 (1.8–2.5)	1.4 (1.2–1.7)	1.5 (1.2–1.9)
Poor mental health	22.9	5.8	4.0 (3.3–4.8)	2.9 (2.3–3.6)	2.3 (1.8–3.0)

Abbreviations: CI, confidence interval; NHANES, National Health and Nutrition Examination Survey.

a Housing insecure participants responded always, usually, or sometimes to the question “How often in the past 12 months would you say you were worried or stressed about having enough money to pay your rent/mortgage?”

b Socioeconomic measures include education, income, and home ownership.

c Demographics include sex, health insurance status (aged 18–65 years), Hispanic ethnicity, age, marital status, veteran status, presence of children in the home, and adverse childhood experiences.

Compared with people who were not housing insecure, respondents who were insecure were about twice as likely to report poor or fair health status (aPR = 1.9), 14 days or more of poor mental health (aPR = 2.3), or poor health limiting daily activity in the past 30 days (aPR = 2.0). A weaker association was found between housing insecurity and 14 days or more in the past 30 of poor physical health (aPR = 1.5).

## Discussion

We found that respondents who were housing insecure were more likely than those who were not to report the following even after adjusting for demographics and socioeconomic measures: delaying doctors’ visits, poor or fair health, and 14 days or more of poor health or mental health limiting daily activity in the past 30 days. This is not the first study to show an association between housing insecurity and health (2–4), but to our knowledge, it is the first to show that such associations exist even after controlling for various socioeconomic and demographic measures. This study also shows the value of using data from both the ACE and Social Context state-added BRFSS modules.

The findings in this report are subject to at least 4 limitations. First, because the BRFSS is a cross-sectional survey, it is not possible to determine if housing insecurity and health outcomes are causally related. Second, the BRFSS excludes participants who are homeless. People who experienced housing insecurity and then became homeless would not be included, perhaps leading to an underestimation of the association between housing insecurity and poorer health. Third, even though possible confounders were controlled for in the model, residual confounding from using categorical variables could still exist, and not all possible confounders could be controlled. Finally, BRFSS data are self-reported and subject to recall and social desirability bias.

This analysis supports a call to action among public health practitioners addressing disparities to focus on social determinants of health risk behaviors and outcomes as barriers for people to achieve optimal health (7,8). The National Prevention Council’s Action Plan, for example, emphasizes that affordable housing can help make healthy lifestyle choices easier (8). Such engagement represents an expansion of public health’s traditional housing-related efforts that focused on environmental health and safety (9,10) and encourages multisector collaboration as well as nuanced approaches toward health equity.
